# Tubulin islands containing slowly hydrolyzable GTP analogs regulate the mechanism and kinetics of microtubule depolymerization

**DOI:** 10.1038/s41598-020-70602-0

**Published:** 2020-08-12

**Authors:** Jonathan A. Bollinger, Zachary I. Imam, Mark J. Stevens, George D. Bachand

**Affiliations:** Center for Integrated Nanotechnologies, Sandia National Laboratories, Albuquerque, NM 87185 USA

**Keywords:** Nanoscale biophysics, Microtubules, Biological physics, Self-assembly

## Abstract

Dynamic instability of microtubules is characterized by stochastically alternating phases of growth and shrinkage and is hypothesized to be controlled by the conformation and nucleotide state of tubulin dimers within the microtubule lattice. Specifically, conformation changes (compression) in the tubulin dimer following the hydrolysis of GTP have been suggested to generate stress and drive depolymerization. In the present study, molecular dynamics simulations were used in tandem with in vitro experiments to investigate changes in depolymerization based on the presence of islands of uncompressed (GMPCPP) dimers in the microtubule lattice. Both methods revealed an exponential decay in the kinetic rate of depolymerization corresponding to the relative level of uncompressed (GMPCPP) dimers, beginning at approximately 20% incorporation. This slowdown was accompanied by a distinct morphological change from unpeeling “ram’s horns” to blunt-ended dissociation at the microtubule end. Collectively these data demonstrated that islands of uncompressed dimers can alter the mechanism and kinetics of depolymerization in a manner consistent with promoting rescue events.

## Introduction

Microtubules are stiff, high aspect-ratio biopolymers integral to many cellular processes in eukaryotic cells and relevant for diverse bioengineering, therapeutic, and materials science applications^1^. Specifically, microtubules are a key driver of organelle transport, cellular motility, mitosis, meiosis, and cytoskeletal structure^2^. Because of their involvement in a wide range of biological functions, the dynamic behavior of microtubules is critical for proper cell function of healthy cells, as well as key therapeutic targets for diseased cells^3^. Likewise, their dynamic and mechanical properties makes microtubules models for biology-inspired materials including synthetic tubules^4^, stimuli-responsive gels^5^, and non-equilibrium liquid–crystal systems^6^. Thus, the fundamental structure–function relationship and behaviors of microtubules have been broadly interesting from both a biological as well as physical perspective.

Microtubules exhibit such diverse functionality largely due to dynamic instability, the stochastic process by which microtubules cycle between periods of slow polymerization (10–50 nm s^−1^) and fast depolymerization (300–500 nm s^−1^)^7,8^. Polymerization or growth proceeds by the addition of GTP-tubulin (*αβ*-tubulin heterodimer bound to GTP) to both ends of the microtubule. Away from the lengthening GTP-tip or “cap,” the GTP is hydrolyzed to GDP with a slight delay^9^, which in turn destabilizes the microtubule lattice^10^. The destabilization of the microtubule lattice has been attributed to a conformational change in the *α*-subunit that occurs following GTP hydrolysis^11^. As long as the rate of GTP-tubulin addition at the end exceeds the rate of hydrolysis, the microtubule maintains the GTP-cap, which is able to stabilize the microtubule lattice. If the hydrolysis rate exceeds the addition rate, the microtubule undergoes rapid depolymerization or shrinkage via distinctive outwardly-peeling “ram’s horns” that release dimers and produce mechanical work^2,12–15^. The unpeeling and ram’s horns morphology are related to the mechanical stress along the protofilaments due to the propagative conformational changes in the tubulin dimer that follow GTP hydrolysis.

The sudden switch from polymerization to depolymerization is defined as microtubule catastrophe, while the switch from depolymerization to polymerization is defined as microtubule rescue^16^. As discussed above, catastrophe has been associated with the loss of the GTP-cap, which is then followed by rapid shrinkage of the microtubule. Rescue events and, more generally, slowdown of depolymerization have been associated with the presence of islands of GTP-tubulin in the lattice^17,18^. The supposition is that these islands may act as vestigial GTP-cap regions that can slow down and/or inhibit depolymerization and thus serve as points for initiating rescue. Tropini et al.^18^ tested this concept by forming sections within a microtubule that contained a mixture of GTP and GMPCPP (guanosine-5′-[(α,β)-methyleno]triphosphate), and evaluated the rate of shortening and frequency of rescue in these sections compared to sections with only GDP tubulin. A significant decrease in the rate of shortening was observed for sections containing GMPCPP, consistent with prior reports^19^, while the frequency of rescue and regrowth correlated with the size and composition of the islands^18^. These findings support the idea that islands of GTP may serve as intrinsic points of microtubule rescue. The underlying mechanism and role of different tubulin states and conformations within the microtubule lattice during depolymerization and rescue, however, remains uncertain.

Here, we used a combination of experiments and molecular dynamics (MD) simulations to examine how dimer conformation impacts the dynamics and morphology of microtubule depolymerization and provides opportunity for microtubule rescue. Specifically, we simulated the depolymerization of coarse-grained models of microtubules comprised of islands with mixtures of dimers possessing different shapes (straight/compressed) and performed corresponding experiments on microtubules that were polymerized to form microtubule with of islands containing GDP and GMPCPP tubulin. We observed the same exponential slowdown in the depolymerization rate in simulation and experiment that scaled with the amount of uncompressed (GMPCPP) tubulin dimers in the lattice, where this slowdown begins at a critical threshold of 20% uncompressed (unhydrolyzed) content. Simulations reveal how this slowdown plays out at the nanoscale: not only is there a change from ram’s horns to more blunt-ended morphologies, but also the global slowdown in depolymerization results from an increased instantaneous end-stabilization that is dependent on the local concentration of uncompressed dimers. Simulations show how the random distribution of unhydrolyzed dimers yields clusters in the lattice that slow the depolymerization rate. In particular, clusters similar to stabilizing caps strongly slow depolymerization, providing both time for rescue and seeds for polymerization. Taken together, the simulations and experiments underline that tubulin shape is a key regulator of microtubule behavior and point to a direct relationship between dimer compression and hydrolysis.

## Results

### Uncompressed/GMPCPP-tubulin dimers slow down and alter the mechanism of catastrophic depolymerization

**Figure 1:**
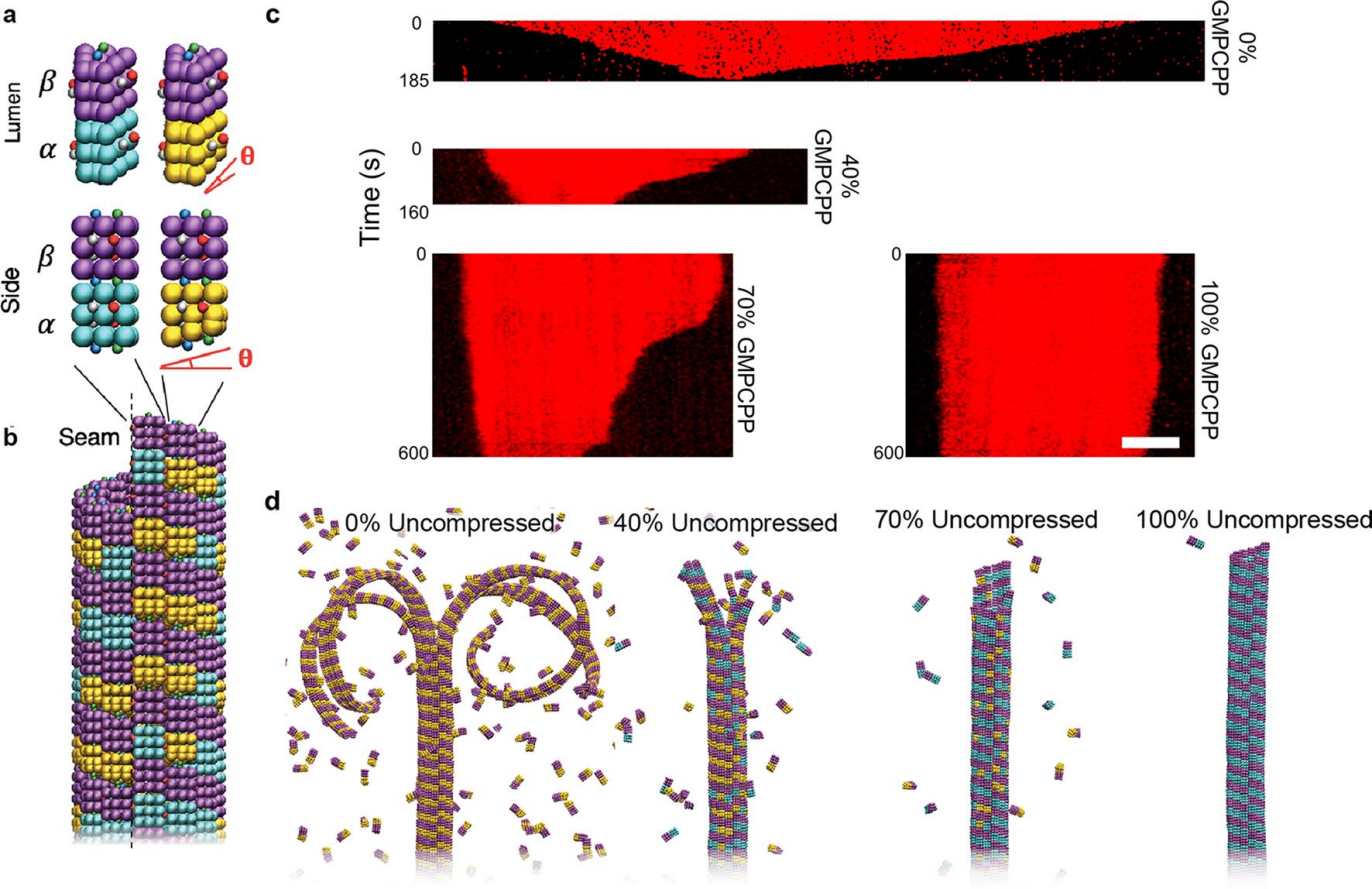
Model and experimental systems for examining microtubule depolymerization dynamics. (**a**) Coarse-grained models of tubulin dimers, which comprise wedge-shaped *α*- (blue, gold) and *β-*subunits (purple) that are either identical in shape (uncompressed dimers, left) or with *α*-subunits that are vertically compressed by angle *θ* (compressed dimers, right). Model wedges include lateral and vertical attractive sites that have color-specific interactions, where lateral sites are vertically offset across the wedges to stabilize the pitch-3 microtubule lattice. (**b**) Top portion (plus-end) of a full model microtubule containing 40% uncompressed dimers prior to depolymerization. Wedges are shaped such that thirteen vertical protofilaments fit in the lattice, representing the typical number of protofilaments in a typical microtubule in vivo. (**c**) Kymographs of TRITC-labeled microtubules with 0%, 40%, 70%, and 100% incorporated GMPCPP. Timescale are included on the left of each kymograph. All microtubules are displayed with the minus-end on the left and plus-end on the right. Scale bar correspond to 2 µm. (**d**) Full model microtubules composed of 0%, 40%, 70%, and 100% uncompressed dimers undergoing depolymerization.

We compared depolymerization rates in simulations with compressed/uncompressed dimers to experimental microtubules with GDP/GMCPP dimers to understand how depolymerization depends on dimer conformation and nucleotide state (Fig. 1). In the MD simulations, the *α*- and *β*-subunits of tubulin were modeled as wedges with attractive points to hold the subunits together and form protofilaments (Fig. 1a), which were assembled into microtubules consisting of thirteen protofilaments (Fig. 1b). The conformational effects of hydrolysis were approximated as a compression of the *α*-subunit, which was previously shown to drive catastrophic depolymerization of model microtubules^20^. For visualization, uncompressed GTP-tubulin dimers were labeled in purple and blue and compressed GDP-tubulin dimers were in purple and yellow (Fig. 1a). Experimentally-derived microtubules were formed by using varying ratios of GTP and GMPCPP (a slowly-hydrolysable GTP analog), the latter of which has been used to inhibit depolymerization and commonly used in microtubule rescue studies^18^. Here, GMPCPP-tubulin dimers are known to adopt a straight uncompressed conformation^11^ and thus serve as analogs of unhydrolyzed GTP-tubulin. Because GTP has a stronger binding affinity, the percentage of GMPCPP incorporation into the microtubules was estimated by correcting for the fourfold difference in affinity between GTP and GMPCPP (derivation and summary table in Supplementary Information)^18^. In addition, there was an underlying assumption in the experimental work that the majority of GTP-tubulin is hydrolyzed to GDP.

The rates of depolymerization observed in simulations and experiments qualitatively displayed an inverse relationship to level of GMPCPP (Fig. 1c,d). As anticipated, microtubules polymerized with 100% GTP depolymerized extremely rapidly, with microtubules completely vanishing from the field of view between 20 and 150 s (Fig. S1). Similarly, rapid depolymerization was observed in the MD simulations for microtubules lacking uncompressed dimers, where the simulated microtubules exhibit the classic ram’s horns morphology, consistent with previous experimental and modeling work. The inclusion of GMPCPP-tubulin resulted in a slowdown in the rate of microtubule depolymerization, as qualitatively observed in fluorescence kymographs (Fig. 1c). For example, microtubules with 40% GMPCPP-tubulin remained in the field of view at times exceeding 400 s, as compared to complete disappearance of most of the positive control (100% GTP) microtubules in less than 100 s. At 70% and 100% GMPCPP tubulin, microtubules remained in the field of view after 600 s (Fig. 1c), displaying only slight decreases in length (100% GMPCPP). Similar results in microtubule depolymerization were observed in MD simulations with reduced rates of depolymerization correlating with increasing levels of uncompressed dimers in the lattice. More importantly, the increase in uncompressed dimers resulted in dramatic changes in morphology of the depolymerizing microtubule. For example, at 70% uncompressed dimers, depolymerizing microtubules no longer exhibited the dramatic ram’s horns morphologies that are indicative of catastrophic depolymerization, but rather adopted blunt morphologies (Fig. 1d). Moreover, disassociation of dimers from these blunt ends was found to be much slower than those dissociating from ram’s horns, which we address in the next section and in the discussion of Fig. 4.

### Slowdown of depolymerization result from increasingly prevalent interruption events

**Figure 2:**
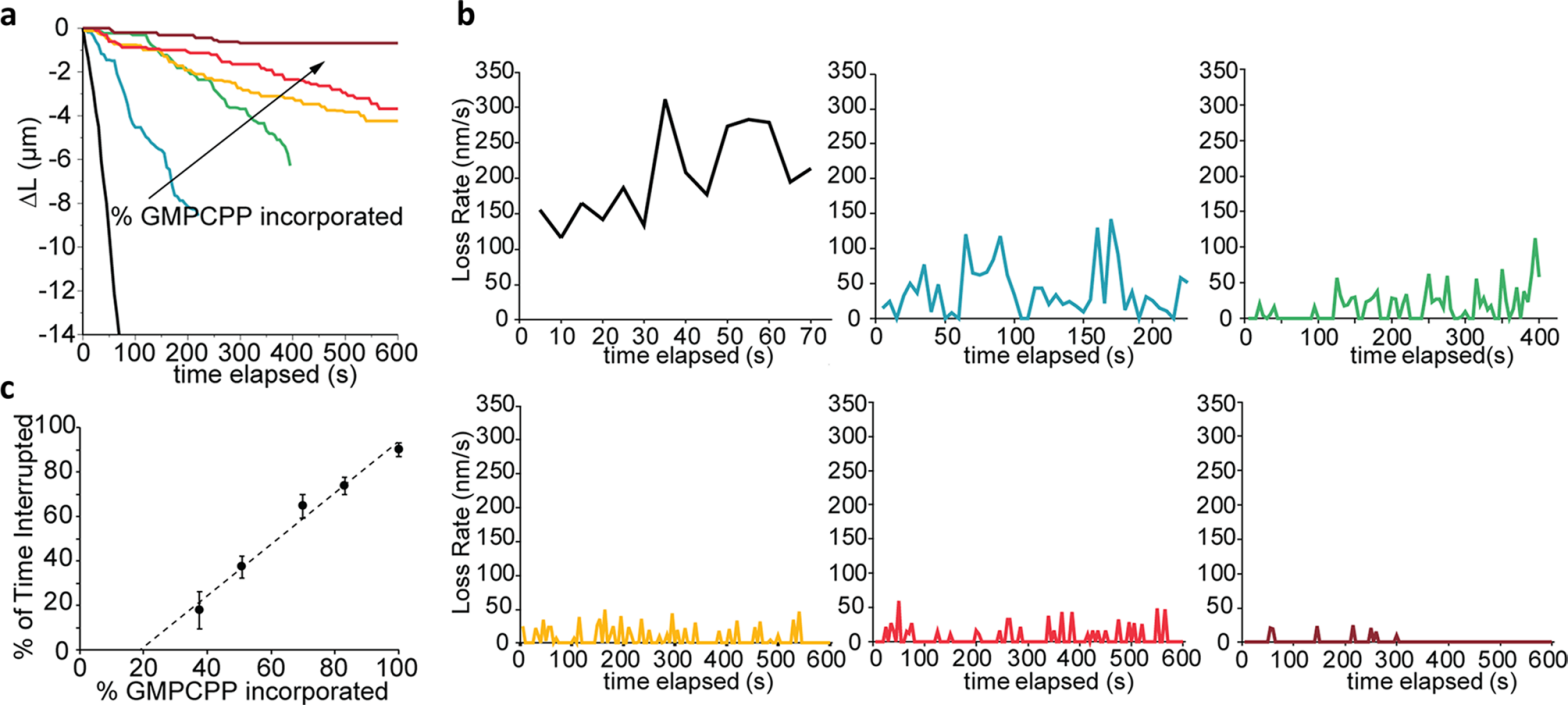
Incorporation of GMPCPP-tubulin decreases microtubule depolymerization rate and increases frequency of interruptions in microtubule depolymerization. (**a**) Net length of microtubule lost during the time of experiments for a representative microtubule for each GMPCPP-tubulin incorporation examined: 0% (black), 40% (blue), 50% (green), 70% (yellow), 80% (red), and 100% (maroon). (**b**) The instantaneous loss rates over the time course of experiments for the representative microtubules corresponding to those in (**a**). (**c**) The average percentage of time microtubules were interrupted as a function of % GMPCPP-tubulin incorporation. Error bars are 95% confidence intervals. Data were fit using linear regression, y = 115.4x − 22.1, R^2^ = 0.983.

The quantitative effect of GMPCPP-tubulin in the microtubule lattice was experimentally characterized by measuring the rate of depolymerization over 10 min as a function of increasing ratios of incorporated GMPCPP-tubulin (i.e., 0%, 40%, 50%, 70%, 80%, and 100%; N = 102 microtubules). Characteristic examples of the change in microtubule length versus time for each ratio are shown in Fig. 2a. As anticipated, the 100% GTP (0% GMPCPP) microtubules depolymerized rapidly (~ 0.243 ± 0.022 µm s^−1^; mean ± standard error of the mean) whereas the addition of GMPCPP-tubulin significantly altered the rate and progression of depolymerization (P > 0.001, Supplementary Table 1), consistent with prior studies^18,19,21,22^. For example, at as low 40% GMPCPP-tubulin, the lifetime of the microtubule increased considerably and included instances in which the microtubule temporarily ceased depolymerization, herein referred to as “interruptions.” The frequency and duration of these interruptions correlated directly with the amount of incorporated GMPCPP-tubulin. Here, the frequency of interruptions increased from an average of 0.6 ± 0.15 interruptions µm^−1^ at 40% GMPCPP to 2.8 ± 0.45 interruptions µm^−1^ at 100% GMPCPP (Supplementary Table S2). Similarly, the average duration of interruptions increased from 7.8 to 46.7 s at 40% and 100% GMPCPP, respectively (Supplementary Fig. S1). These data support the idea GMPCPP-tubulin form islands in the microtubule lattice, and the hypothesis that these islands significantly alter the kinetics of depolymerization.

Additional changes in depolymerization were noted by measuring the instantaneous loss rate over time for the different populations of microtubules (Fig. 2b). For the control (100% GTP), the rate of depolymerization maintained a relatively constant rate (100–300 nm s^−1^) over time with minor fluctuations. In contrast, the instantaneous loss rate of 40% GMPCPP microtubules decreased significantly below that of 100% GTP control microtubules (Fig. 2b). This observation agreed with the MD simulations in which tubulin dissociation was slower when the microtubule adopted a blunt end as opposed to ram’s horns. The data clearly demonstrated that the decreased instantaneous loss rates and increased number of interruptions correlated with the increased amount of GMPCPP tubulin incorporated into the microtubule lattice. Thus, we attribute the decreased depolymerization rates (Fig. 2b) to the slower dissociation of tubulin dimers from the blunt microtubules ends along with an increased frequency of interrupted depolymerization events due to the presence of GMPCPP islands within the microtubule lattice.

The increased frequency of interruptions is particularly relevant to microtubule dynamics as these events represent opportunities for microtubule rescue to occur. As such, the percentage of time that a microtubule spent in the interrupted state was measured and observed to increase linearly with the percentage of incorporated GMPCPP (R^2^ = 0.983; Fig. 2c). Extrapolating from this regression, we estimated that this effect should become evident with as low as 20% GMPCPP incorporation in the microtubule lattice. Unfortunately, experiments to test this effect were unsuccessful as microtubules polymerized with 20% GMPCPP (and without paclitaxel) fully depolymerized before they could be observed by microscopy.

### Simulations and experiments supports correlation between hydrolysis and dimer compression

**Figure 3:**
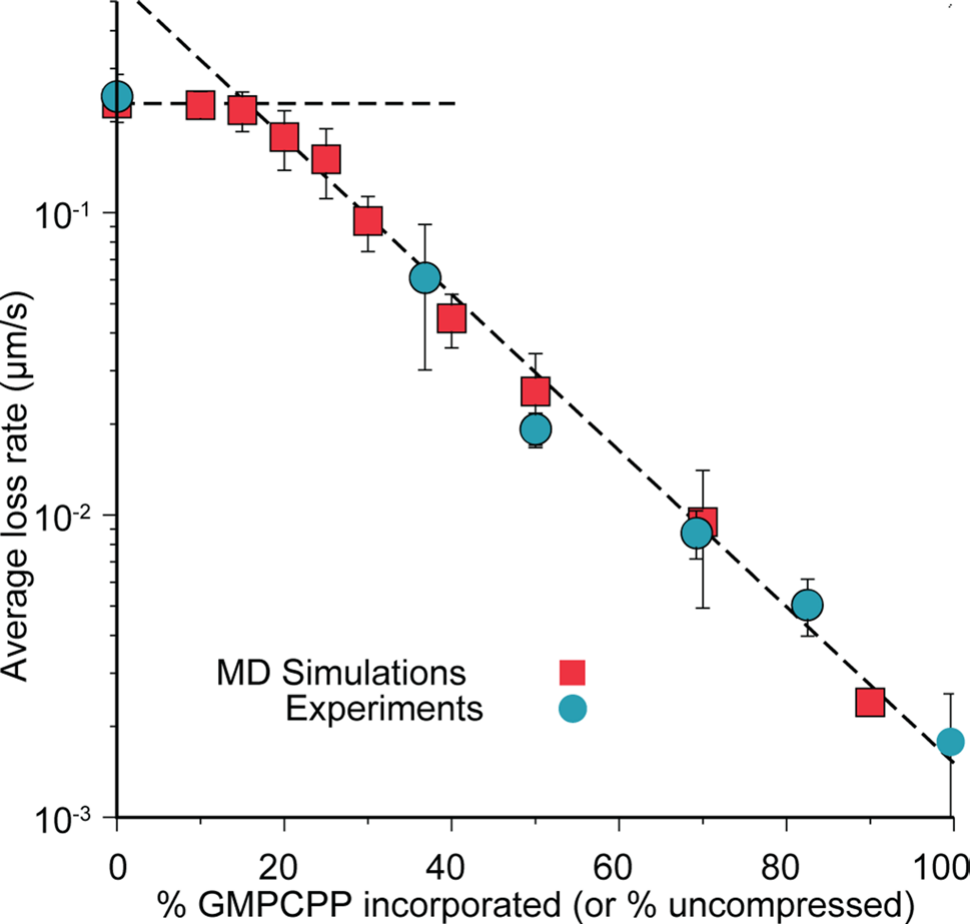
Depolymerization rates versus microtubule composition. Average rates of loss in microtubule length versus % GMPCPP incorporated (experiments, blue circles) and % uncompressed dimers (simulations, red squares). Horizontal dashed line corresponds to 0.240 µm s^−1^. Negatively sloped dashed line corresponds to y = (0.60 µm s^−1^)exp[− 0*.*060*x*] where *x* is % GMPCPP incorporated (uncompressed dimers).

MD simulations confirmed the observed decrease in depolymerization rate as a function of increasing levels of uncompressed tubulin (GMPCPP tubulin in experiments). The simulated depolymerization rates were measured using a similar methodology as in the experiments and confirmed the exponential slowdown of the depolymerization rate based on the increased percentage of uncompressed dimers. Plotting the experimental and simulation data demonstrated strong agreement between these two approaches (Fig. 3). Of particular note, the depolymerization rate of the microtubules in MD simulations was not affected until around 15–20% incorporation, which was consistent with the extrapolation from the linear regression (Fig. 2c) that suggested a critical concentration of 20% GMPCCP in terms of affecting depolymerization. This agreement between the MD simulations and experiments underlines a one-to-one correspondence between nucleotide state and dimer compression. In turn, the onset of exponential slowdown meant that, for example, at 30% uncompressed, the rate of microtubule depolymerization had already fallen by 60% compared to 0% in both simulations and experiments. Furthermore, by 50% uncompressed, depolymerization had been almost completely interrupted, decreasing in rate by approximately 90%.

### Instantaneous depolymerization rates and mechanisms depend on local lattice composition

**Figure 4:**
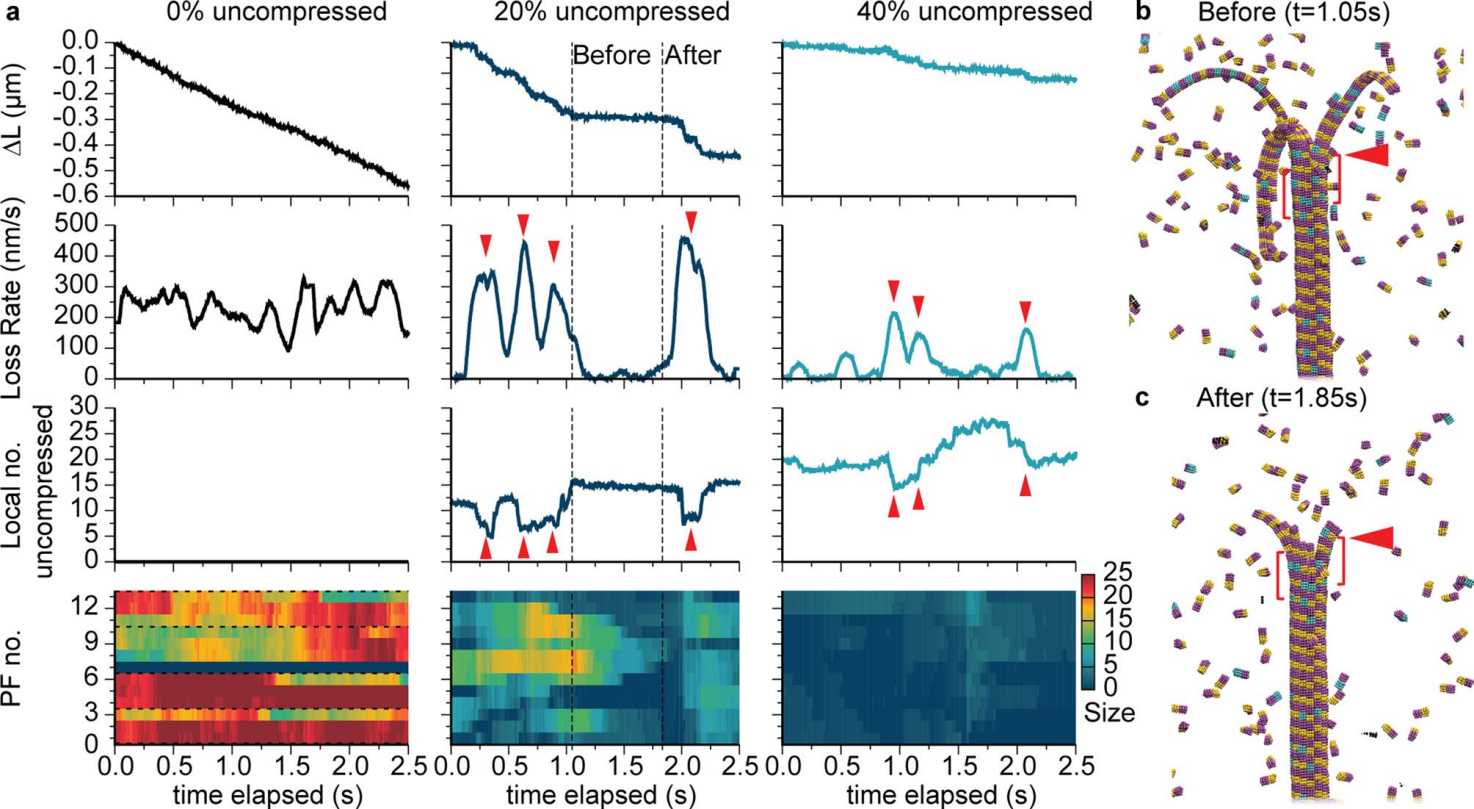
Instantaneous depolymerization rates and mechanisms as a function of microtubule composition from simulations. (**a**) Net length of microtubule lost ∆*L*, depolymerization loss rate, local number of uncompressed dimers, and heat maps indicating sizes of exposed protofilaments as a function of simulation time elapsed. Local numbers of uncompressed dimers are based on the first full three dimer rows after the ram’s horns. Exposed protofilament lengths are measured starting from the last intact row of the microtubule; horizontal dashed lines indicate observed boundaries of ram’s horns. Downward (upward) red triangles highlight instances of large depolymerization rates (low numbers of uncompressed dimers) for 20% and 40% uncompressed. (**b,c**) Model microtubule configurations before and after an interruption for 20% uncompressed, corresponding to the dashed lines in (**a**). Red triangles in both panels indicate the intact portion of the microtubule at 1.05 s that defines the three-row region for counting uncompressed dimers shown in brackets.

Because of our access to the MD simulations, we were able to consider how variations in the instantaneous rate of depolymerization (e.g., interruptions) related to the underlying local composition of compressed versus uncompressed dimers. As shown in Fig. 4a, for 0% uncompressed, where there was no variation in local shape composition, we observed essentially steady-state loss in microtubule length with stochastic variations in instantaneous rate ranging between approximately 200 and 300 nm s^−1^ about the average loss rate of 240 nm s^−1^. However, by 20% uncompressed dimers, there were clear periods of interruption, with the rate of depolymerization exhibiting corresponding fluctuations between fast (comparable to rates at 0% uncompressed dimers) and slow (near zero) loss. Here, rates during stretches of depolymerization were often faster than the base rates seen at 0% uncompressed, typical of depolymerization start-up beginning from an intact model microtubule. By 40%, rates were always lower than that typically observed at 0%, with various interruptions occurring throughout time. As shown in Fig. 4a, we generally observed that these instantaneous depolymerization rates roughly *mirrored* the number of locally uncompressed dimers present ahead of the depolymerization front (within a window of three dimer rows, as highlighted in Fig. 4b,c). In other words, periods of relatively fast depolymerization occurred when the proximal portion of the microtubule lattice contained relatively few uncompressed dimers, and vice versa. Collectively, these observations suggested that tubulin shape was a controlling factor globally (as in Fig. 3) because it was a controlling factor locally.

The mechanism by which depolymerization instantaneously proceeds as a function of microtubule composition was quantified by calculating the exposed lengths of protofilaments (i.e., lengths of protofilaments starting from the last intact row of the microtubule) over time in the MD simulations. As shown in the bottom portion of Fig. 4, at 0% uncompressed dimers, the exposed protofilament lengths were large, ranging from 12–25 dimers and corresponding to ram’s horns that were 2–3 protofilaments in width (as also shown in Fig. 1.) (In this case, an exception was the protofilament labeled 7, which was passed back and forth rapidly between its neighboring protofilaments while being depleted, and thus having an exposed length of approximately zero.) There was typically one protofilament undergoing this process in all cases, though its position varied.) In contrast, with as little as 20% uncompressed dimers, ram’s horns developed occasionally and corresponded to periods of fast depolymerization (similar to the rates for 0% uncompressed dimers). These ram’s horns were periodically depleted when the depolymerization front approached a lattice region containing more uncompressed dimers, as exemplified by the before-and-after states shown in Fig. 4b,c. By 40% uncompressed dimers, only short exposed protofilaments (five dimers or less) were observed, with the depolymerization mechanism characterized by fraying. Thus, we conclude that the presence of clusters of uncompressed dimers disrupts the normal mechanism of catastrophic depolymerization by limiting the formation of high-strained ram’s horns that the microtubule end.

### Slowdown commences due to the prevalence of large clusters of uncompressed dimers

**Figure 5:**
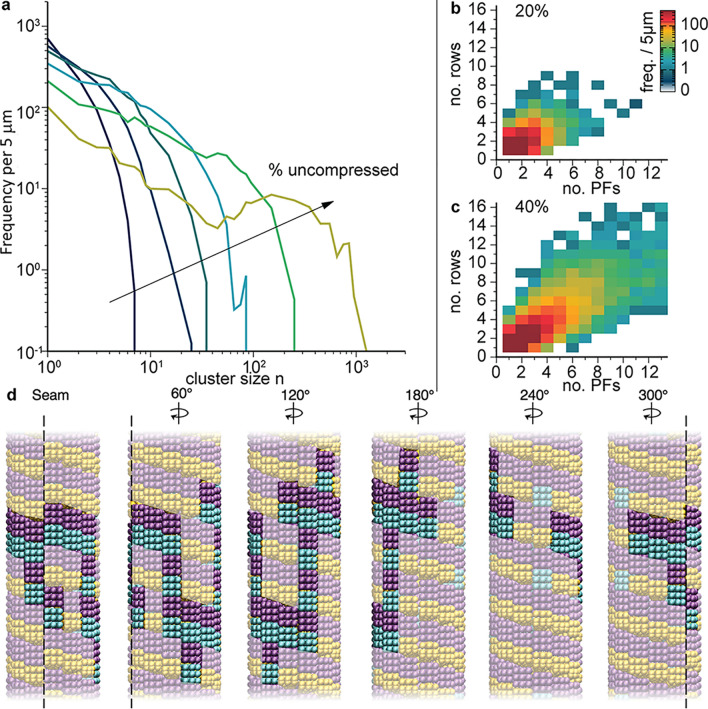
Clusters of uncompressed dimers as a function of microtubule composition. (**a)** Histogram *h*(n) of cluster size distribution per 5 µm weighted by size *n* to make data at large *n* visible in plot. Histograms are for 10, 20, 30, 40, 50, and 60% (dark blue to light green) uncompressed microtubules. Uncompressed dimers were considered part of the same cluster if they share at least one vertical or horizontal subunit bond. (b, c) Heat maps of frequency of cluster footprints observed for 20% and 40% uncompressed, respectively, where footprints are given in terms of numbers of dimer rows and protofilaments spanned by the cluster. (d) Images at different rotations of the largest cluster observed for 20% uncompressed, comprising 21 dimers and having a footprint spanning 6 dimer rows and 11 protofilaments.

To understand the onset and progression of the depolymerization slowdown, we next considered the spatial distribution of uncompressed dimers and how they clustered as a function of microtubule composition. We calculated the histogram *h*(*n*) of uncompressed dimer clusters sizes *n* per 5 µm, as described in detail in the Methods section, and plotted *n h*(*n*) to make the frequency of large *n* clusters visible (Fig. 5a). Below 20% uncompressed only small clusters of connected uncompressed dimers (*n* < 10) occurred in the randomly distributed dimers (Fig. 5a). At this level of uncompressed dimers, slowdown of depolymerization was not observed as the ram’s horns could proceed around and/or through these clusters. However, by 20% uncompressed, clusters with sizes of twenty or more uncompressed dimers occurred in the lattices, with footprints spanning up to several dimer rows and ten or more protofilaments (Fig. 5b). Though relatively infrequent, such clusters led to interruptions as they were comparable in size to stabilizing plus-end caps, which prevent depolymerization. These clusters resemble that shown in Fig. 5d, which spans eleven protofilaments and six dimer rows. By 40% uncompressed dimers, the largest clusters approached 100 dimers in size and clusters routinely reached thirteen protofilaments in width (Fig. 5c), leading to significant slowdown and disruption of the ram’s horns mechanism (Fig. 4a). Finally, by 50–60% uncompressed, clusters were frequently hundreds of dimers in size, with local maxima developing in the cluster size distribution (Fig. 5a), indicating the onset of lateral cluster percolation. Such large clusters led to nearly complete (approximately 90%) slowdown and blunt-ended depolymerization as they provided stabilized microtubule plus-ends.

## Discussion

Microtubule dynamic instability is characterized by stochastic, alternating phases of growth (polymerization) and shrinkage (depolymerization), which allows eukaryotic cells to rapidly reorganize as physiological needs dictate. The loss of the GTP-tubulin cap at the growing end of the microtubule has been shown to trigger catastrophe, defined as the transition from growth to shrinkage (depolymerization). What is less understood is what prompts the transition from shrinkage to growth, known as rescue. Work by Dimitrov et al. demonstrated that remnants of GTP-tubulin (i.e., islands) are embedded within the lattice of the microtubule polymerized both in vivo and in vitro, and that these islands are frequently associated with the initiation of rescue events^17^. This rescue initiation mechanism is supported by the work of Tropini et al.^18^ in which predefined sections of GMPCPP-tubulin were shown to reproducibly induce rescue events. In this work, a general decrease in the rate of shortening was also noted for sections containing GMPCPP as compared to sections polymerized with GTP^18^, which agrees with prior studies with GMPCPP microtubules^19,21,22^.

In the present work, we provide a thorough analysis of how GMPCPP islands regulate the kinetics and mechanism of microtubule shrinkage in a manner that promote rescue events. Specifically, we show through experiments and simulations that islands of GMPCPP-tubulin may be stochastically formed during the polymerization process, and that the size, shape, and geometry of these island significantly impact microtubule depolymerization. For example, significant decreases in microtubule depolymerization rates and increases in the frequency and time spent in an interrupted state were strongly correlated with the percentage of incorporated GMPCPP-tubulin (Fig. 2). These data suggest that the presence of GMPCPP-tubulin islands in the microtubule lattice disrupts the mechanism of depolymerization in a manner that provides frequent temporal pauses (interruptions), which in principle allow GTP-tubulin to bind to the end and reestablish growth. For example, at 40% incorporated GMPCPP-tubulin, microtubules experience an interruption every 1.2 µm, each lasting an average of approximately 7.8 s. Extrapolation of the experimental data (Fig. 2c) suggests that as little as 20% GMPCPP-tubulin incorporation may be sufficient to disrupt microtubule depolymerization and provide opportunities for rescue events to occur. This value is supported by MD simulations, where a critical threshold for affecting the average loss rate incorporation was observed at the inclusion of 20% uncompressed dimers (Fig. 3).

The simulation results indicate that the tubulin dimer shape is a key regulator of depolymerization, where the determining feature of the model is the conformational change from uncompressed to compressed tubulin. Therefore, the depolymerization trends observed in our simulations emerge from treating the effect of GTP hydrolysis of *α*-tubulin as a shape change in the simulated tubulin. This decision for the model was inspired by the results from high-resolution cryo-EM that demonstrated hydrolysis of the E-site nucleotide induces compression in the *α*-tubulin subunit^11,23^. In our experiments, the depolymerization properties correlated strongly with the incorporation of the slowly-hydrolysable GTP analog, GMPCPP, which has been shown to maintain a straight (uncompressed) conformation similar to that observed for GTP-tubulin prior to lattice hydrolysis^11^. Thus, our work supports a mechanistic basis for stabilizing microtubules against depolymerization: counteracting the effect of conformational changes in tubulin. Stabilizing agents including paclitaxel and Tau^24^ have been shown to inhibit these conformational changes and prevent microtubule depolymerization. We speculate that stabilizing the conformation of tubulin may also be the mechanism by which certain microtubule-associated proteins (MAPs) such as CLASP^25,26^ and CLIP-170 prevent depolymerization^27–29^. Our results suggest that these proteins may provide stabilization by acting on multiple tubulin dimers in a microtubule. Thus, at these specific locations the stabilizing proteins may act as an “effective” GTP island that slows depolymerization and promotes rescue in a similar fashion as true GTP-tubulin islands.

In light of our results, the necessary features to model the rich dynamics of microtubules are made clearer. First, it is essential to model the full dimensionality (i.e., 3D) of each tubulin within a microtubule. Treating the microtubule in this way reveals the interacting dynamics of tubulins in each protofilament. This dimensionality is further necessary to produce the appropriate curvature for ram’s horns developed during depolymerization, which are a key feature following catastrophe. Importantly, the 3D changes to ram’s horns morphology as uncompressed dimers are incorporated are vital for full understanding of the impact of GTP islands on the mechanism of microtubule depolymerization. Secondly, the interaction strengths of the individual tubulins are also important. In our model, the interaction strengths are constrained to a narrow range of values that yield catastrophic depolymerization with the long ram’s horns found experimentally^30^. With these features, the 3D coarse-grained model may be used to investigate the dynamic morphology of microtubule ends, the effect of different GTP island clusters, and the kinetics of depolymerization. The strong agreement with the depolymerization of experimental microtubules implies that the choices of attraction strengths and especially the dimer conformational change capture the essential physics of tubulin. Thus, the model for tubulin examined here should prove useful in future simulation studies of nucleation, growth, and rescue—microtubule behaviors which have been historically difficult to model due to the length and time scales involved in their development.

Overall, our work demonstrates that islands of uncompressed/GMPCPP tubulin dimers within the microtubule lattice are capable of altering the kinetics of depolymerization and morphology of microtubule ends in a manner that is consistent with promoting rescue events. While GTP islands have been observed in vivo and in vitro*,* the prevalence and regulatory nature of these islands remain uncertain. Processes such as GDP-to-GTP exchange^31^ and microtubule self-renewal^32^, and the action of severing enzymes (e.g., spastin and katanin)^33^ likely play critical roles in the formation of these islands, and must be considered in future experiments and simulations. In addition, experimental work and molecular dynamics simulations could be further adapted to include proteins (e.g., MAPs and CLASPs), osmolytes and/or divalent metal ions that have been observed to affect microtubule stability^34,35^. Such investigations could yield critical insights into how cells regulate the dynamic instability of microtubule filaments as well as the dynamics of other cytoskeletal filaments.

## Methods

### Chemicals and buffers

PIPES (Piperazine-N,N-bis(2-ethanesulfonic acid)), MgCl_2_, EGTA, KOH, GTP, paclitaxel (Taxol), casein, AMP-PNP (*β*,*γ*-imidoadenosine 5′-triphosphate), glucose oxidase, catalase, D-glucose, 6-hydroxy-2,5,7,8-tetramethylchroman-2-carboxylic acid, DTT, and Trolox were purchased from Sigma-Aldrich. Unlabeled and TRITC (tetramethylrhodamine) lyophilized porcine brain tubulin were purchased from Cytoskeleton, Inc. The slowly hydrolysable GTP analogue, GMPCPP, was purchased from Jena Biosciences.

### Microtubule preparation

TRITC labeled and unlabeled tubulin in a 1:6 molar ratio was suspended in BRB80 buffer (80 mM PIPES, 1 mM MgCl_2_, and 1 mM EGTA adjusted to pH 6.9 with KOH) containing 1 mM nucleotide at a concentration of 16 µM and then polymerized for 30 min at 37 °C. After polymerization microtubules formed from 100% GTP were stabilized by diluting to 0.8 µM with BRB80T, BRB80 buffer containing 1 µM paclitaxel. Microtubules formed from 40%, 50%, 70%, 80%, and 100% GMPCPP were stabilized by diluting to 0.8 µM with BRB80. All samples were stored at room temperature.

### Flow cell preparation

A 5 mm × 20 mm × 0.15 flow cell was created on a glass slide using double-sided tape and a glass coverslip. The motor protein kinesin (KIF5B) was used to immobilize microtubules to the coverslip surface for imaging. To inhibit motor actuation, a nonhydrolyzable ATP analog, AMP-PNP, was used. Kinesin was expressed and purified using established protocols^36^. The flow cell was filled with 1 µM kinesin in the buffer BRB80CA, BRB80 buffer containing 0.2 mg mL^−1^ casein, 1 mM AMP-PNP, and 1 mM Trolox. After a 5-min incubation, 20 *µ*L of the microtubule solution was introduced into the channel. Following another 5-min incubation, the channel was washed three times with imaging buffer, BRB80 buffer containing, 0.2 mg mL^−1^ casein, 1 mM AMP-PNP, 1 mM Trolox, 0.02 mg mL^−1^ glucose oxidase, 0.008 mg mL^−1^ catalase, 20 mM D-glucose, and 1 mM DTT. The flow cell was immediately sealed with Valap and imaged as described below.

### Microscopy and data analysis

Fluorescence imaging was performed on an IX-81 Olympus microscope with 100X/1.4 N.A. oil immersion objective, Semrock Brightline Pinkel DA/FI/TR/Cy5/Cy75X-A000 filter set, 1.5 ND filter, and Orca Flash 4.0 digital camera. For microtubule depolymerization experiments, images were acquired at 5-s intervals for a total duration of 10 min. The endpoints of the microtubule filaments were tracked in ImageJ to measure the microtubule depolymerization rates for each experimental group.

### Coarse-grained tubulin models

We adopted a previously-described model of *αβ*-tubulin where *α*- and *β*-subunits were represented by wedges decorated with short-range attractive sites that represent regions for tubulin-tubulin binding^20^. The wedges were composites of 27 beads that interacted with the analogous beads of other wedges via the cut and shifted Lennard Jones pair potential *U*_LJ_(*r*) =  4*ε*[(*σ/r*)^12^ (*σ/r*)^6^]+ *ε* out to cutoff distance 2^1*/*6^*σ*, where ε and *σ* were the characteristic energy scale and bead diameter, respectively. Each wedge included eight additional attractive sites divided into four pairs (two lateral and two vertical), where lateral pairs were vertically-offset to stabilize the pitch-3 chiral lattice of microtubules when in register. Pairs of attractive sites interacted with their counterparts on the opposite surfaces (i.e., top-to-bottom, left-to-right) of another wedge via the potential *U*_A_(*r*) =  − *A*[1 + cos(*πr/r*_c_)] out to cutoff distance *r*_c_ = 0*.*75*σ*. Throughout, we set the prefactors for lateral and vertical interactions between dimers to *A*_L_ = 1*.*4*k*_b_*T* and *A*_V_ = 4*.*8*k*_b_*T*, respectively, which together were known to reproduce aspects of the microtubule dynamic instability given appropriate choices of compression angle *θ* (discussed below)^30^. For vertical interactions between the *α* and *β* subunits of the same dimer, we set *A*_*intra*_= 8*.*0 *k*_b_*T* to prevent self-dissociation.

The *αβ*-tubulin dimers in the absence of hydrolysis were represented by two identically-shaped wedge monomers (Fig. 1a), while dimers subjected to hydrolysis were represented by replacing the *α*-subunit with one compressed by angle *θ* in the vertical direction to approximate the conformational effects of hydrolysis observed in experiments^11,23^. Due to the placement of binding sites, the uncompressed dimers energetically favored straight protofilament configurations while the compressed dimers energetically favored curved protofilaments. Throughout, we fixed *θ* = 15° to mimic the relative compressive allostery observed by Alushin et al.^37^ This results in our hydrolyzed protofilaments having a radius curvature of 28 nm matching experimental values^19,38,39^ and a longitudinal compaction of 2%, when comparing pure GDP-microtubules to pure GTP-microtubules^20^.

### Molecular dynamics simulations

Model microtubule structural dynamics were characterized as a function of composition by pre-building tubules out of compressed and uncompressed dimers in various ratios. During this building process, uncompressed or compressed dimers were randomly added as set by the probability of uncompressed incorporation. In all cases here, we examined model microtubules in isolation and after all possible hydrolysis has taken place (i.e., we did not model individual hydrolysis events). The minus-ends of all model microtubules were tethered to prevent tubule drift by fixing the initial positions of all beads in the two bottom-most dimer rows of the microtubule lattice. For systems that were 25% or less uncompressed, we simulated microtubules that were 160 dimer rows (equivalent to roughly 1.4 µm) in length. For systems that were 30%-50% uncompressed, we simulated microtubules that were 80 dimer rows (0.72 µm) in length. For all other systems, we simulated microtubules that were 40 dimer rows (0.36 µm) in length. For all simulations, microtubules were initialized in a vertical configuration with all horizontal attractive sites in-register. For each compression level, five independently generated microtubules were simulated to calculate depolymerization kinetics. For the clustering analysis, at each compression level, we independently generated 20 microtubules that were 555 dimer rows (2.4 μm) in length. We scanned these microtubules to generate cumulative histograms *h*(*n*) of the cluster sizes *n* present across all 20 microtubules (i.e., cluster frequencies over 20 × 2.4 μm of microtubule length). Since the count depends on the microtubule length, we chose 5 μm as the length and these histograms *h*(*n*) are the average counts per 5 μm of microtubule length.

MD simulations were performed using the LAMMPS package^40^ with a time-step of *δt* = 0*.*005*τ*, where $^{T} = \sigma (m/\varepsilon )^{1/2}$ and *m* was the reference mass of one bead. The temperature was fixed at $T = 1.0\varepsilon /k_{b}$ (where *k*_b_ is Boltzmann’s constant) using a Langevin thermostat with damping constant 1.0*τ*^−1^. The wedge-shaped subunits (including attractive sites) were effectively treated as self-contained rigid objects by connecting all beads within the subunits via harmonic potentials with spring constant *K* = 500*k*_b_*T/σ*^2^. To map LJ timescales to real units for comparisons with experiments, we matched the depolymerization rate calculated for 0% uncompressed microtubules to the depolymerization rate of the pure GTP-tubulin microtubules, resulting in a conversion of 3 × 10^6^*τ* ≡ 1 s.

## Supplementary information

Supplementary information.

## References

[1] Bachand GD, Spoerke ED, Stevens MJ (2015). Microtubule-based nanomaterials: exploiting nature's dynamic biopolymers. Biotechnol. Bioeng..

[2] Howard J, Hyman A (2003). Dynamics and mechanics of the microtubule plus end. Nature.

[3] Jordan MA, Wilson L (2004). Microtubules as a target for anticancer drugs. Nat. Rev. Cancer.

[4] Wagenbauer KF, Sigl C, Dietz H (2017). Gigadalton-scale shape-programmable DNA assemblies. Nature.

[5] Boekhoven J, Hendriksen WE, Koper GJM, Eelkema R, van Esch JH (2015). Transient assembly of active materials fueled by a chemical reaction. Science.

[6] Wu K-T (2017). Transition from turbulent to coherent flows in confined three-dimensional active fluids. Science.

[7] Desai A, Mitchison TJ (1997). Microtubule depolymerization dynamics. Annu. Rev. Cell Dev. Biol..

[8] Mitchison T, Kirschner M (1984). Dynamic instability of microtubule growth. Nature.

[9] Caplow M, Shanks J (1996). Evidence that a single monolayer tubulin-GTP cap is both necessary and sufficient to stabilize microtubules. Mol. Biol. Cell.

[10] Maurer SP, Fourniol FJ, Bohner G, Moores CA, Surrey T (2012). EBs Recognize a nucleotide-dependent structural cap at growing microtubule ends. Cell.

[11] Alushin GM (2014). High-resolution microtubule structures reveal the structural transitions in \(αβ\)-tubulin upon GTP hydrolysis. Cell.

[12] Chrétien D, Fuller SD, Karsenti E (1995). Structure of growing microtubule ends: two-dimensional sheets close into tubes at variable rates. J. Cell Biol..

[13] Kueh HY, Mitchison TJ (2009). Structural plasticity in actin and tubulin polymer dynamics. Science.

[14] Mandelkow EM, Mandelkow E, Milligan RA (1991). Microtubule dynamics and microtubule caps: a time-resolved cryo-electron microscopy study. J. Cell Biol..

[15] Westermann S (2006). The Dam1 kinetochore ring complex moves processively on depolymerizing microtubule ends. Nature.

[16] Gardner, M. K., Zanic, M. & Howard, J. in *Current Opinion in Cell Biology* (2013). 10.1016/j.ceb.2012.09.006 PMC3556214 23092753

[17] Dimitrov A (2008). Detection of GTP-tubulin conformation in vivo reveals a role for GTP remnants in microtubule rescues. Science.

[18] Tropini C, Roth EA, Zanic M, Gardner MK, Howard J (2012). Islands containing slowly hydrolyzable GTP analogs promote microtubule rescues. PLoS ONE.

[19] Müller-Reichert T (1998). Structural changes at microtubule ends accompanying GTP hydrolysis: information from a slowly hydrolyzable analogue of GTP, guanylyl (alpha, beta)methylenediphosphonate. Proc. Natl. Acad. Sci. USA.

[20] Bollinger JA, Stevens MJ (2018). Catastrophic depolymerization of microtubules driven by subunit shape change. Soft Matter.

[21] Mickey B, Howard J (1995). Rigidity of microtubules is increased by stabilizing agents. J. Cell. Biol..

[22] Walker RA (1988). Dynamic instability of individual microtubules analyzed by video light-microscopy—rate constants and transition frequencies. J. Cell. Biol..

[23] Zhang R, Alushin GM, Brown A, Nogales E (2015). Mechanistic origin of microtubule dynamic instability and its modulation by EB proteins. Cell.

[24] Shigematsu H (2018). Structural insight into microtubule stabilization and kinesin inhibition by Tau family MAPs. J. Cell Biol..

[25] Al-Bassam J (2010). CLASP Promotes microtubule rescue by recruiting tubulin dimers to the microtubule. Dev. Cell.

[26] Lawrence EJ, Zanic M (2019). Rescuing microtubules from the brink of catastrophe: CLASPs lead the way. Curr. Opin. Cell Biol..

[27] Aher A, Akhmanova A (2018). Tipping microtubule dynamics, one protofilament at a time. Curr. Opin. Cell Biol..

[28] de Forges H (2016). Localized mechanical stress promotes microtubule rescue. Curr. Biol..

[29] Komarova YA, Akhmanova AS, Kojima S-I, Galjart N, Borisy GG (2002). Cytoplasmic linker proteins promote microtubule rescue in vivo. J. Cell Biol..

[30] Bollinger JA, Stevens MJ (2019). Diverse balances of tubulin interactions and shape change drive and interrupt depolymerization. Soft Matter.

[31] Piedra F-A (2016). GDP-to-GTP exchange on the microtubule end can contribute to the frequency of catastrophe. Mol. Biol. Cell.

[32] Schaedel L (2019). Lattice defects induce microtubule self-renewal. Nat. Phys..

[33] Vemu A (2018). Severing enzymes amplify microtubule arrays through lattice GTP-tubulin incorporation. Science.

[34] Bachand GD, Jain R, Ko R, Bouxsein NF, Vandelinder V (2018). Inhibition of microtubule depolymerization by osmolytes. Biomacromol.

[35] O’Brien, E. T., Salmon, E. D. & Erickson, H. P. How calcium causes microtubule depolymerization. *Cell Motil. Cytoskeleton* **36**, 125–135. 10.1002/(SICI)1097-0169(1997)36:2<125::AID-CM3>3.0.CO;2-8 (1997). 10.1002/(SICI)1097-0169(1997)36:2<125::AID-CM3>3.0.CO;2-8 9015201

[36] Bachand GD (2004). Assembly and transport of nanocrystal CdSe quantum dot nanocomposites using microtubules and kinesin motor proteins. Nano Lett..

[37] Alushin GM (2014). High-Resolution microtubule structures reveal the structural transitions in αβ-Tubulin upon GTP hydrolysis. Cell.

[38] Elie-Caille C (2007). Straight GDP-tubulin protofilaments form in the presence of Taxol. Curr. Biol..

[39] Wang H-W, Nogales E (2005). Nucleotide-dependent bending flexibility of tubulin regulates microtubule assembly. Nature.

[40] Plimpton S (1995). Fast parallel algorithms for short-range molecular dynamics. J. Comput. Phys..

