# Supplementary material for: Tubulin islands containing slowly hydrolyzable GTP analogs regulate the mechanism and kinetics of microtubule depolymerization
Source: Sci Rep. 2020 Aug 12;10:13661. doi: 10.1038/s41598-020-70602-0 (PMC7423891; doi:10.1038/s41598-020-70602-0)
Supplement: Supplementary file 1 — Supplementary information. [file 41598_2020_70602_MOESM1_ESM.pdf]

Tubulin islands containing slowly hydrolysable GTP analogs regulate the mechanism and kinetics of microtubule depolymerization

Jonathan A. Bollinger, Zachary I. Imam, Mark J. Stevens, and George D. Bachand

Center for Integrated Nanotechnologies, Sandia National Laboratories, Albuquerque NM 87185, USA

Supplementary Information

Calculation of % GMPCPP Incorporated into Microtubules.
We established estimated the percentage of GMPCPP incorporated into microtubules using a previously published calculation.[1] To complete this calculation, we need to know the affinity of GTP for tubulin relative to GMPCPP:

$$\frac{K^*_{GMPCPP}}{K^*_{GTP}} = 3.87$$

The ratio of the dissociation constants (i.e. the affinity of GTP for tubulin relative to GMPCPP).
Once the ratio is known, we can calculate the percentage of GMPCPP incorporated into microtubules using the following equation:

$$\% \, GMPCPP_{Incorporated} = \frac{\frac{[GMPCPP_{Bulk}]}{[GTP_{Bulk}]}}{\left(\frac{[GMPCPP_{Bulk}]}{[GTP_{Bulk}]} + \frac{K^*_{GMPCPP}}{K^*_{GTP}}\right)}$$

Where $[GMPCPP_{Bulk}]$ is the concentration of GMPCPP in solution and $[GTP_{Bulk}]$ is the concentration of GTP in solution.

$GMPCPP_{Bulk}$	$\frac{GMPCPP_{Bulk}}{GTP_{Bulk}}$	$\% \, GMPCPP_{Incorporated}$
0	0.00	0.000
70	2.33	0.376
80	4.00	0.508
90	9.00	0.699
95	18.90	0.830
100	1000.00	1.000

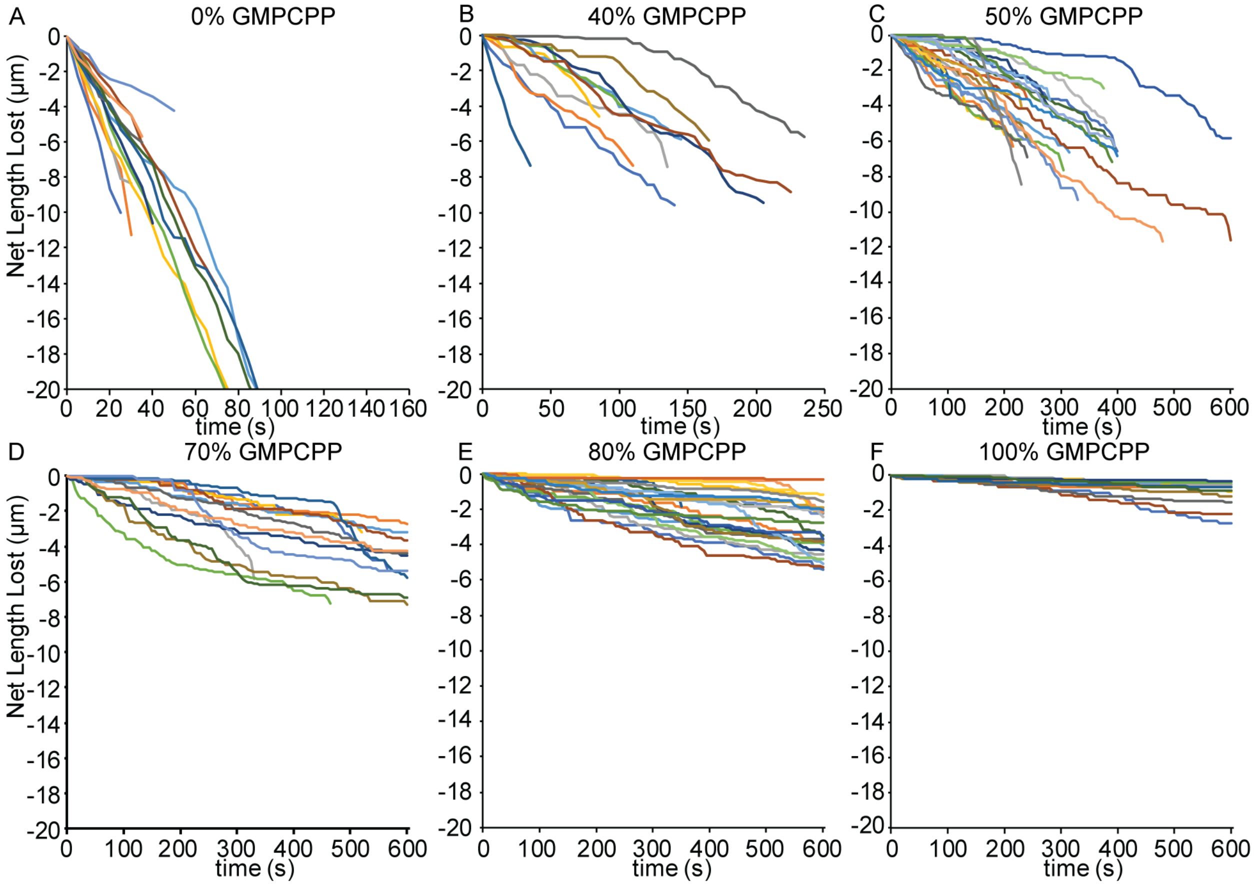

Supplementary Figure 1. Microtubule depolymerization over time. Here are line plots of the net length lost in microns of experimental microtubules over the time course of the experiment for microtubules comprised of 0% GMPCPP (A), 40% GMPCPP (B), 50% GMPCPP (C), 70% GMPCPP (D), 80% GMPCPP (E), and 100% GMPCPP (F).

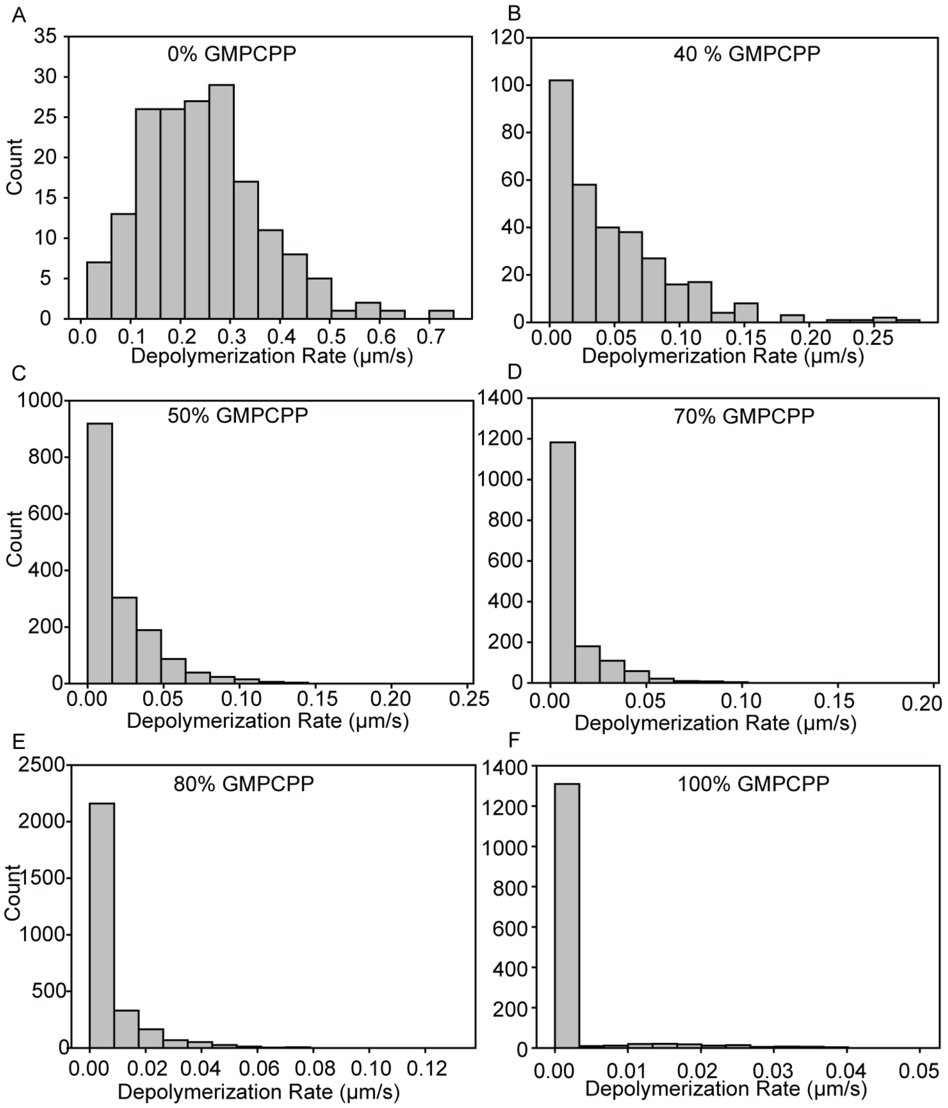

Supplementary Figure 2. Histograms of microtubule depolymerization rates for each condition. The range of instantaneous depolymerization rates for all microtubules measured by fluorescence microscopy for microtubules comprised of 0% GMPCPP (A), 40% GMPCPP (B), 50% GMPCPP (C), 70% GMPCPP (D), 80% GMPCPP (E), and 100% GMPCPP (F).

Supplementary Table 1. Summary statistics with experimental microtubule depolymerization rates. One-way analysis of variance and Fisher LSD Pairwise Multiple Comparison of rates of microtubule depolymerization at varying levels of incorporated GMPCPP.

Group Name	Mean (μm s^{-1})	Std Dev	SEM
Control (0%)	0.243	0.0933	0.0220
40% GMPCPP	0.061	0.0497	0.0150
50% GMPCPP	0.019	0.0050	0.0010
70% GMPCPP	0.009	0.0037	0.0010
80% GMPCPP	0.005	0.0049	0.0010
100% GMPCPP	0.002	0.0001	0.0001

One-Way Analysis of Variance

Source of Variation	DF	SS	MS	F	P
Between Groups	5	0.786	0.157	86.681	<0.001
Residual	96	0.174	0.00181		
Total	101	0.961			

Pairwise Multiple Comparison Procedures (Fisher LSD Method)

Comparison	Diff of Means	LSD (α=0.050)	P
Control vs. 40%	0.182	0.0324	<0.001
Control vs. 50%	0.224	0.0261	<0.001
Control vs. 70%	0.234	0.0301	<0.001
Control vs. 80%	0.238	0.0264	<0.001
Control vs. 100%	0.241	0.0333	<0.001

Supplementary Table 2. Summary of experimental microtubules interruptions and statistical analyses. The interruptions per µm, interruption duration, and percentage of time interrupted for each GMPCPP microtubule condition tested. Mean ± standard error of the mean.

% GMPCPP	Interruptions per µm	Interruption Durations	% Time Interrupted
Control (0%)	0 ± 0.00	0 ± 0.00	0 ± 0.00
40% GMPCPP	0.59 ± 0.14	7.79 ± 0.87	17.9 ± 4.15
50% GMPCPP	2.01 ± 0.22	10.9 ± 0.60	37.2 ± 2.40
70% GMPCPP	3.36 ± 0.46	15.7 ± 0.90	64.9 ± 2.60
80% GMPCPP	3.04 ± 0.27	20.4 ± 1.27	73.6 ± 1.90
100% GMPCPP	2.85 ± 0.48	46.7 ± 2.27	90.1 ± 1.50

Interruptions per µm

Group Name	Mean	Std Dev	SEM
Control (0%)	0.000	0.000	0.000
40% GMPCPP	0.590	0.464	0.140
50% GMPCPP	2.010	1.075	0.215
70% GMPCPP	3.360	1.702	0.455
80% GMPCPP	3.040	1.298	0.265
100% GMPCPP	2.850	1.502	0.475

Source of Variation	DF	SS	MS	F	P
Between Groups	5	152.934	30.587	23.187	<0.001
Residual	96	126.640	1.319		
Total	101	279.574			

Comparison	Diff of Means	LSD(α=0.050)	P
Control vs. 40%	0.590	0.873	0.183
Control vs. 50%	2.010	0.705	<0.001
Control vs. 70%	3.360	0.812	<0.001
Control vs. 80%	3.040	0.711	<0.001
Control vs. 100%	2.850	0.899	<0.001

Interruption Duration

Control (0%)	0.000	0.000	0.000
40% GMPCPP	7.790	2.885	0.870
50% GMPCPP	10.900	3.000	0.600
70% GMPCPP	15.700	3.349	0.895
80% GMPCPP	20.400	6.197	1.265
100% GMPCPP	46.700	7.163	2.265

Source of Variation	DF	SS	MS	F	P
Between Groups	5	15797.469	3159.494	169.439	<0.001
Residual	96	1790.090	18.647		
Total	101	17587.559			

Comparison	Diff of Means	LSD(α=0.050)	P
Control vs. 40%	7.790	3.280	<0.001
Control vs. 50%	10.900	2.650	<0.001
Control vs. 70%	15.700	3.054	<0.001
Control vs. 80%	20.400	2.673	<0.001
Control vs. 100%	46.700	3.381	<0.001

% Time Interrupted

Group Name	Mean	Std Dev	SEM
Control (0%)	0.000	0.000	0.000
40% GMPCPP	17.900	13.764	4.150
50% GMPCPP	37.200	12.000	2.400
70% GMPCPP	64.900	9.728	2.600
80% GMPCPP	73.600	9.308	1.900
100% GMPCPP	90.100	4.743	1.500

Source of Variation	DF	SS	MS	F	P
Between Groups	5	91439.825	18287.965	200.050	<0.001
Residual	96	8776.015	91.417		
Total	101	100215.840			

Comparison	Diff of Means	LSD(α=0.050)	P
Control vs. 40%	17.900	7.263	<0.001
Control vs. 50%	37.200	5.867	<0.001
Control vs. 70%	64.900	6.763	<0.001
Control vs. 80%	73.600	5.918	<0.001
Control vs. 100%	90.100	7.485	<0.001

References Cites

1 Tropini, C., Roth, E. A., Zanic, M., Gardner, M. K. & Howard, J. Islands Containing Slowly Hydrolyzable GTP Analogs Promote Microtubule Rescues. *PLoS One* **7**, e30103, doi:10.1371/journal.pone.0030103 (2012).
